# S02 Exploring equity and social inclusion in national PA policy documents and policy monitoring

**DOI:** 10.1093/eurpub/ckae114.201

**Published:** 2024-09-26

**Authors:** Sonja Kahlmeier

**Affiliations:** Swiss Distance University of Applied Science, Department of Health

## Abstract

Equity and social inclusion are prerequisites for ethical public health services provision. In the field of physical activity promotion, ensuring wide and equitable access to PA infrastructure and services has been recognized as a modality to address social inequities in health.

However, it is still not clear how these desiderates translate in national PA policies.

This symposium aims to cover this gap by presenting aspects related to equity and social inclusion in national and transnational policy documents and policy monitoring and evaluation frameworks.

Systematic literature review, thematic policy documents analysis, stakeholders' consultations and other quantitative and qualitative methods are employed in the four studies presented in the symposium.

